# Estimated functional space of centric condyle positions in temporomandibular joints of asymptomatic individuals using MRI

**DOI:** 10.1038/s41598-019-52081-0

**Published:** 2019-10-30

**Authors:** Aleš Čelar, André Gahleitner, Stefan Lettner, Josef Freudenthaler

**Affiliations:** Medical University of Vienna, University Clinic of Dentistry, Sensengasse 2a, 1090 Wien, Austria

**Keywords:** Bone, Oral anatomy

## Abstract

Magnetic resonance imaging (MRI) studies on centric condyle positions lack 3D comparisons of guided and unguided methods, which are used for accomplishing centric relation reference positions. The purpose of this study was to describe the space, in which mandibular condyles are placed *in vivo* by dental intercuspation, Dawson’s bimanual manipulation, and neuromuscular position. Twenty asymptomatic individuals aged 23 to 37 years underwent separate bite registrations using bimanual manipulation and the unguided neuromuscular technique. Subsequent 3-Tesla MRI scans of both temporomandibular joints yielded 3D data of the most superior condylar points at maximum intercuspation and both centric relation positions. We found concentric condyle positions in maximum intercuspation but considerable variation of condyle position after bimanual manipulation and neuromuscular technique. Their 95% predictive confidence ellipses overlapped substantially and created a space of reference positions. Its smallest volume averaged 2 mm^3^ for a minimal convex hull (95% confidence interval 1.1–3.2) and 3.5 mm^3^ for a minimal ellipsoid hull (95% confidence interval 1.8–5.4). Visualized *in vivo* by MRI, condyle positions at bimanual manipulation and neuromuscular position were not predictable and showed substantial variation in asymptomatic subjects. Clinicians should be aware of the functional space and its effect on dental intercuspation.

## Introduction

Assessment of dental intercuspation (intercuspal position, ICP) is part of the clinical examination of the craniomandibular system. Next to ICP, a centric mandibular reference position (RP) independent of tooth contact is expected to correctly place mandibular condyles against articular discs and eminences. RPs may be essential for extensive prosthetic procedures, comprehensive orthodontics, and orthognathic surgery. Anteroposterior RP-ICP shifts exceeding 1–2 mm and any lateral discrepancy must be documented according to orthodontic diagnostic standards^1^.

**Figure 1:**
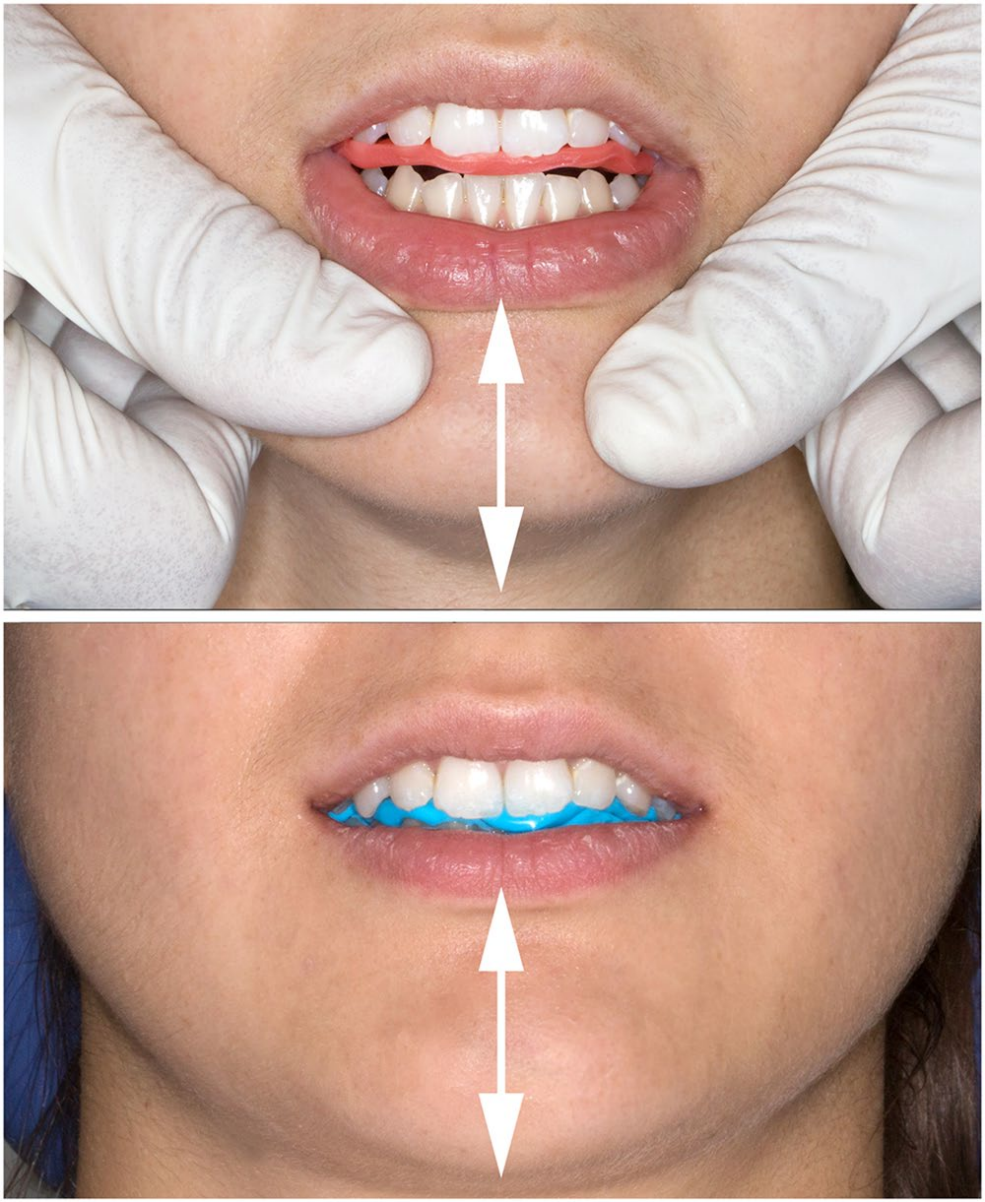
Operator-guided maneuver of bimanual manipulation (BM) with interocclusal wax registration (above). Patient-generated neuromuscular (NM) position registered with polyvinyl siloxane (below). The depicted individual did not participate in the study.

The determination of RP has been under debate for decades. Canonical definitions of centric relation changed from *most retruded* to *anterior-superior*^2^. Among several techniques for the registration of RP, bimanual manipulation (BM) and the neuromuscular method (NM) represent two different concepts: BM is operator-guided^3–5^ whereas NM is entirely patient-generated without manual influence of the examiner^6–9^ (Fig. 1).

In the past most knowledge about condylar position has originated from roentgenographic studies (transcranial head film, orthopantomogram, conventional and computed tomographies). These direct methods do not characterize soft tissues and cartilage, same as scintigraphy. Indirect methods include articulators, condylar position indicators, or recorded hinge axis movements but their representation of the actual anatomical condyle position may remain uncertain. For comprehension of the effects of BM and NM on condyle and disk positions *in vivo*, magnetic resonance imaging (MRI) fulfills the requirement of a direct method for hard and soft tissues. Although a diagnostic gold standard, research on centric condyle positions with MRI has been rare. Two MRI studies analyzed ICP and guided RPs^10,11^ but without MRI visualization of condyle positions achieved by an unguided technique. Hence the aim of the present paper was to directly visualize and evaluate centric condyle position in 3D with MRI when 3 jaw positions are used: ICP, BM, and NM. The null-hypothesis stated no difference between BM and NM in 3 spatial planes.

## Results

The radiologist diagnosed all temporomandibular joints (TMJs) to be free from pathologic conditions. Measurements of the bite registration thickness at the first molars averaged 2.0 ± 0.4 mm for BM and 2.5 ± 0.9 mm for NM registration media.

### Error of method

Determination of the most superior condylar points in MRI scans on 3 different days yielded an average error of 0.57 mm (minimum 0.04 mm, maximum 0.91 mm). Intraclass correlation coefficients (ICCs) showed good intrarater (0.92, 95% confidence interval [CI] from 0.85 to 0.95) and interrater reliabilities (0.91, 95% CI from 0.83 to 0.95).

### Differences between BM, NM, ICP

In the sagittal plane the average BM condyle position was most superior (left TMJ) or most anterior-superior (right TMJ) and the average NM condyle positions were most anterior-inferior. Sagittal differences between BM and NM came to 0.39 ± 0.64 mm (right) and 0.72 ± 1.02 mm (left). Vertically, ICP was located between BM and NM, NM differed vertically from BM 1.39 ± 1.27 mm (right) and 1.26 ± 1.35 mm (left).

Mediolaterally, the BM-NM differences were 0.05 ± 0.64 mm (right) and 0.02 ± 0.54 mm (left). On both sides, BM and NM differed significantly in sagittal (p = 0.002) and vertical (p < 0.001) directions but not in transverse direction, thus rejecting the former hypothesis.

**Figure 2:**
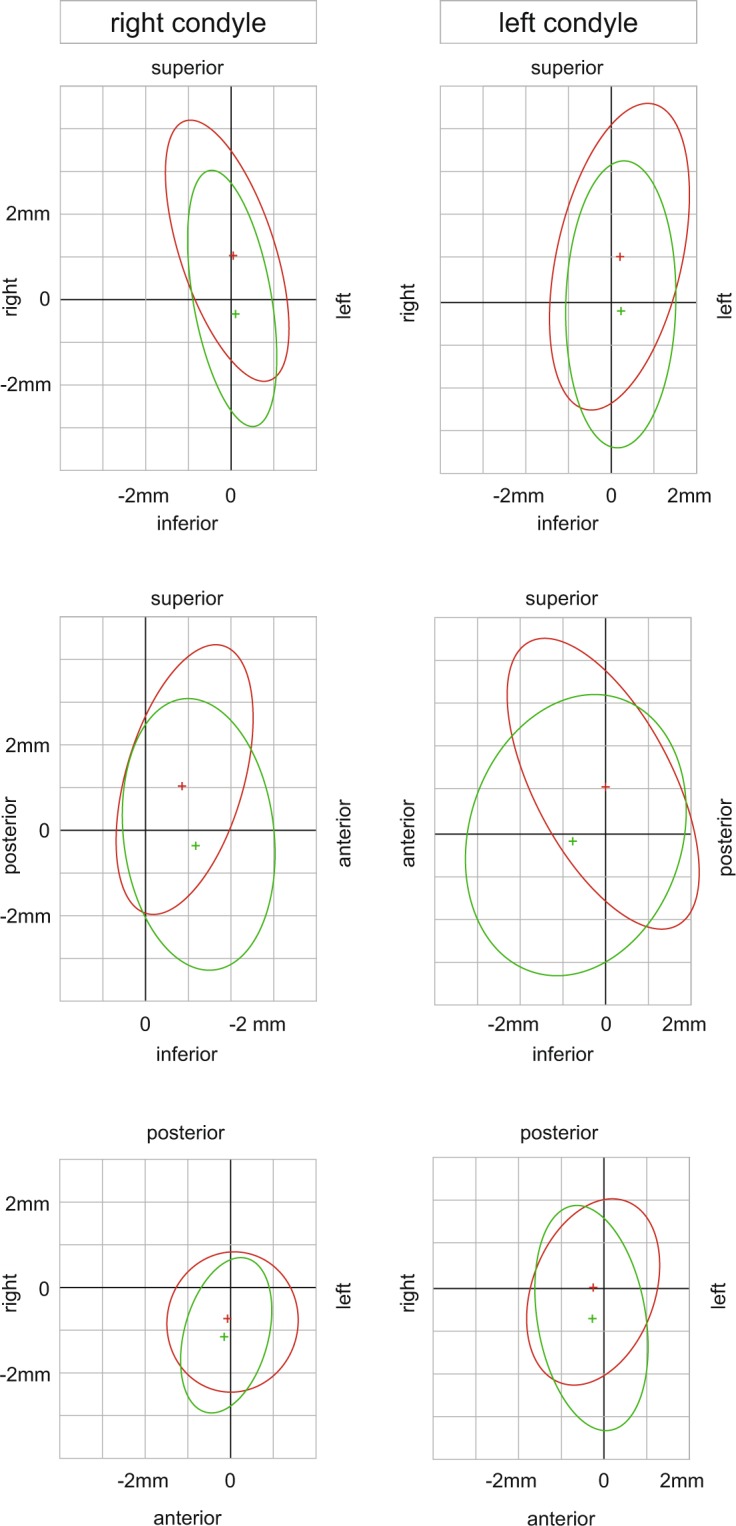
Mean (cross) and 95% predictive confidence ellipses of bimanual manipulation (red) and neuromuscular position (green) related to maximum intercuspation (zero of coordinate system). Graphs on millimeter grids represent frontal, sagittal, and horizontal planes in top-down order.

Sagittally, ICP was more posterior than the average BM on the right but identical with BM on the left side. The 95% confidence ellipses showed considerable variation and BM-NM overlaps in all planes of space (Fig. 2). The orientation of these ellipses was similar on both sides in the sagittal plane. Almost 2/3 of the BM data and nearly half of the NM data were cranial to ICP. NM data scattered more forward downward than BM data.

In the frontal plane the BM and NM ellipses showed considerable overlap. BM condyle positions scattered approximately 1 mm more cranially with small transverse differences. Frontal plane data ellipses were oriented slightly oblique except the left side NM ellipse, which showed clearly vertical orientation. NM produced less transverse deviation of data than BM.

In the horizontal plane the average BM condyle position was 0.42 mm more posterior than NM on the right and 0.65 mm more posterior on left side.

Table 1 shows the spatial distances ICP-BM and ICP-NM descriptively. Results from a logarithmic link model showed that the 3D Euclidean distances between BM and NM averaged 1.37 mm, their 95% CI ranged from 0.95 to 1.79. Table 2 displays the distances ICP-BM and ICP-NM in 2D for the sagittal, horizontal, and transverse planes descriptively. The horizontal plane showed the greatest difference of means and standard deviations between BM and NM.

**Table 1:** 3D distances between intercuspal position (ICP) and bimanually manipulated (BM) and unguided, neuromuscular (NM) condyle positions in millimeters. SD standard deviation, Q1 25% quantile, Q3 75% quantile, Min minimum, Max maximum.

		Mean	SD	Min	Q1	Median	Q3	Max	N
ICP-BM	Right	2.02	1.17	0.50	1.31	1.78	2.60	4.75	20
	Left	1.92	0.96	0.43	1.32	1.79	2.28	4.23	20
ICP-NM	Right	1.91	1.21	0.27	1.18	1.72	2.29	5.74	20
	Left	1.84	0.90	0.68	1.27	1.55	2.24	3.84	20

**Table 2:** Plane projections: Differences between intercuspal position (ICP) and bimanually manipulated (BM) or neuromuscular (NM) condyle positions in millimeters. SD standard deviation, Q1 25% quantile, Q3 75% quantile, Min minimum, Max maximum.

			Mean	SD	Min	Q1	Median	Q3	Max	N
Anterior-Posterior	ICP-BM	Right	−0.77	0.78	−2.30	−1.19	−0.80	−0.44	1.24	20
		Left	0.01	1.03	−2.19	−0.57	−0.28	0.49	2.30	20
	ICP-NM	Right	−1.17	0.73	−2.51	−1.64	−1.19	−0.59	−0.10	20
		Left	−0.71	1.12	−2.88	−1.25	−0.51	−0.04	1.22	20
Horizontal	ICP-BM	Right	1.03	1.66	−4.09	0.37	1.06	1.74	3.77	20
		Left	1.02	1.47	−1.22	−0.04	1.17	2.00	3.62	20
	ICP-NM	Right	−0.37	1.71	−5.38	−1.16	−0.06	0.78	1.71	20
		Left	−0.25	1.47	−3.61	−0.97	0.01	0.81	1.72	20
Left-Right	ICP-BM	Right	0.05	0.77	−1.10	−0.33	−0.10	0.49	2.41	20
		Left	0.22	0.66	−1.15	−0.17	0.04	0.80	1.33	20
	ICP-NM	Right	0.10	0.52	−0.69	−0.20	0.02	0.30	1.63	20
		Left	0.25	0.55	−0.87	−0.10	0.05	0.66	1.25	20

The estimated volume of a minimal functional space occupied by ICP, BM, and NM yielded a minimal convex hull of 2.01 ± 2.46 mm^3^ (95% CI 1.09–3.22) and an ellipsoid hull of 3.47 ± 4.22 mm^3^ (95% CI 1.82–5.39).

## Discussion

The final sample size (n = 20) agrees with Kandasamy *et al*.’s study^11^ on 2 types of manual guidance for achieving centric relation condyle positions. Guided by a power analysis, Kandasamy *et al*. investigated 19 subjects. In our study, all participants did not show signs or symptoms of TMD and clearly met our intention of evaluating asymptomatic individuals exclusively. We considered signs and symptoms of the craniomandibular system as confounders, leading to higher variability of data. Risk factors as articular disc displacement and degenerative joint disease become more likely with increasing age^12–14^. In regard of TMD, young adulthood lends itself to assess asymptomatic physiological conditions for permissible generalisation of experimental outcomes.

In our study the condyles of healthy individuals were centered in the glenoid fossae when in ICP. Applying BM or NM, the direction of condylar shift from ICP was not predictable. Guided positions were significantly more posterior and superior to unguided ones and the latter showed equal or greater variability. In the transversal plane, differences between BM or NM and ICP followed much more a vertical pattern than deviation to left or right. We found a vertical orientation of the NM confidence ellipses while the BM confidence ellipses were slightly oblique. Effects of manual guidance, TMJ morphology, and elasticity of the mandible under load can explain the distribution of BM data. Left-right asymmetries of BM and NM may originate from muscular asymmetry, chewing pattern, asymmetric condylar axes^11,15^, and facial asymmetry^16^. Considering BM and NM, our *in vivo* results question a distinct centered condylar position. The MRI data indicate a space instead.

Lack of concentric condyle position has been observed in asymptomatic human TMJs, too^10,11,17^. Using indirect methods such as articulators, unguided centric relation techniques showed higher variability than guided ones^8,9^. The variability primarily originated from patient condition and to a lesser extent from different operators or 3 different time points^9^. Having used identical guided and unguided techniques as Čelar *et al*.^9^, the confidence ellipses of our MRI data and those generated in an electronic condylar position indicator^9^ were quite analogous in spite of different asymptomatic subjects. We attribute the similarity of confidence ellipses of both studies to a primarily “patient-based” source of variation of BM and NM condyle positions. Because of this resemblance, we hypothesize a similar long-term reproducibility of the present study’s data although not having tested it. Our instant repetitions of BM and NM represent routine clinical verifications.

Diurnal inconsistency of centric relation can add fluctuation and represents a circumscribed degree of freedom of the condyle in centric position^18^. As shown by ultrasound jaw tracking, the mode of bite registration significantly influenced condyle position in asymptomatic individuals^19^. Altogether, variation of centric condylar positions indicated a functional area of RP intermaxillary relations^20^.

For estimation of the dimension of a 3D functional space *in vivo*, we combined guided and unguided MRI condyle position data of our sample. The volume of a minimal convex hull estimate averaged 2 mm^3^, the estimated minimal ellipsoid hull volume 3.5 mm^3^. Both shapes showed substantial standard deviations. The extent of mandibular opening necessary to avoid tooth contact and leave room for bite registration material can also affect condyle position^21^ and subsequently this volume. However, we speculate that healthy individuals will tolerate the leeway of condylar position within few mm^3^. Further studies are needed to define limits of functional space dimensions.

In terms of physiology, condylar concentricity itself did not to warrant a diagnosis of TMJ health^10^. Cone-beam tomographies of samples with and without disc displacement also showed high variability of condylar position in young adults^22^. Data of the anterior disk displacement group did not substantiate higher and more posterior condyle positions than those of the group without disk displacement^22^.

Some authors questioned the tenet of specific centric relation concepts^2,23^ or the possibility of changing condyle position by manipulation^10^. Investigating ICP, retruded centric relation, and Roth power centric relation with MRI, Kandasamy *et al*.^11^ described large standard deviations for their measurements without statistically significant differences. Positioning of condyles by manual mandibular guidance into certain sites of the fossae appeared unrealistic^11^. Consequently Kandasamy *et al*. doubted efforts of producing dentitions, which fit conceptual condyle positions^11^. Using BM and NM in our study showed comparable outcome in spite of different techniques and measuring point definitions.

The question whether guided or unguided techniques are appropriate remains unreciprocated to some degree. Clinicians have divergent opinions about TMJ function and evaluation^24,25^ but pronounced lateral or sagittal RP-ICP shifts of the mandible may indicate a risk factor for craniomandibular dysfunction^26–28^. Small condylar dimension, shape, and inclination predispose to disc displacement^29,30^ and in our opinion to the variation of condylar position. It appears reasonable that asymptomatic individuals with complete natural dentitions will accept self-determined mandibular postures as ICP and NM. This approach remains unclear for patients with already existing restorations or need for them. Guided techniques are useful in detecting actively protruded mandibular postures in Angle class II patients or for establishing an articular reference in class III malocclusion. Overall, RPs maintain their importance and should be registered after examination of the craniomandibular system^1^. However, their scatter within a functional space should be borne in mind.

Limitations of our study may arise from the MRI set-up, sustained jaw postures and immobilization during MRI. Minor body movements due to breathing or jaw opening for incorporation of the bite registrations are additional issues. ICP and positions without tooth contact differ in vertical jaw relationship and may exhibit unequal condylar rotation. Our MRI measuring points were not hinge axis points. Diverse condyle rotation can alter the location of the measuring point on the condylar outline on one hand. On the other hand, hinge axis points will also scatter if condylar rotation and translation occur simultaneously. Further limitations involve long-term reproducibility of BM and NM positions of repeated MRI exams. This issue requires further study.

In conclusion MRIs of TMJs of asymptomatic individuals showed concentric condyle positions in maximum intercuspation whereas no distinct condyle position was found for bimanual manipulation and neuromuscular position. Considerable variation of both latter positions doubted punctilious placing of mandibular condyles in the glenoid fossa and indicated a functional space of reference instead. It connotes multiple diagnostic and therapeutic positions within approximately 5 mm^3^. Clinicians should reflect this quantity and its effect on dental occlusion when making use of RPs in clinical practice.

### Subjects, materials and methods

We conducted this study with approval of the university ethics commission (#ECS1438/2015) in accordance with the ethical standards of the 1964 Declaration of Helsinki and its later amendments. Complying with the STROBE guidelines for reporting observational studies (http://www.equator-network.org/reporting-guidelines/strobe/) we recruited 29 healthy Caucasian volunteer dental students without history of temporomandibular dysfunction via institutional blackboard. Of the initially recruited 29 subjects, 7 individuals showed clinical signs of disc displacement or masticatory muscle pain and were excluded from the study same as 2 individuals with intrauterine metal coils. The final sample consisted of 20 asymptomatic individuals aged from 23 to 37 years, 10 women (mean 26.7 ± 2.4 years) and 10 men (27 ± 3.3 years). This sample size agreed with a former similar study^11^. Every individual included in the study signed an informed consent form after thorough instruction. The person shown in Fig. 1 did not participate in the study but gave written consent for photography and publication.

Participants met following inclusion criteria: asymptomatic TMJs without noise in history and clinical examination, mouth opening of at least 40 mm as well as lateral and protrusive mandibular movements of ≥ 8 mm in clinical examination, complete permanent dentition without consideration of third molars (clinical examination), and unmistakable ICP as judged on dental casts after removal of artefacts (Die-Keen, Heraeus Kulzer, South Bend, IN, USA). Casts had been obtained from alginate impressions (Tetrachrom, Kaniedenta, Herford, Germany).

Anamnestic exclusion criteria were TMJ clicking or crepitus, myofascial pain in the craniomandibular system, systemic muscle disease, neurological disease, connective tissue disease, fibromyalgia, rheumatoid arthritis, orthodontic treatment during the last five years, use of anxiolytic medication, intrauterine contraceptive device, pregnancy, claustrophobia, psychic disease, history of facial trauma or facial pain or TMJ ankylosis. Criteria for exclusion by clinical examination were TMJ clicking or crepitus, myofascial pain, pain on muscle palpation, loss of force during isometric tension, acute or chronic facial pain, recent facial trauma, capsulitis, synovitis, arthritis, limited mandibular movement, and mandibular side shift.

### Sequence of examinations

Two colleagues, who are named in the acknowledgement, screened all candidates’ histories concerning the aforementioned conditions as well as allergy and medication. The colleagues also palpated each candidate’s masticatory muscles and TMJs on pain, clicking, or crepitus. They measured maximum mandibular opening and lateral movements. Having passed the initial triage, candidate participants were offered an appointment for repeated clinical examination of their asymptomatic state by a specialist in clinical diagnosis of TMD. We arranged the MRI scans 2–3 hours after the second clinical exam.

One author (A.C.), experienced in diagnosis and treatment of patients with temporomandibular disorders for 25 years, performed clinical examinations, *i.e*. the manual structural analysis according to Bumann and Lotzmann^7^. A.C. evaluated mandibular movement capacity, unguided and manipulated mandibular protrusion/retrusion (dynamic compression and dynamic translations). He palpated the temporal muscles, medial pterygoid, lateral pterygoid, anterior digastric, posterior digastric, suprahyoid and infrahyoid muscles, superficial and deep masseter muscles, and the trapezoids. Examination on capsulitis and synovitis included tractions of the mandible in posterior, postero-superior, superior, medial, medio-superior, latero-posterior, latero-postero-superior, anterior, and inferior directions. Loss of muscle force and onset of muscular pain were tested during 20 seconds of isometric closure against cotton rolls. Pain, fatigue, or loss of force were also evaluated during maintaining 4 mm left and 4 mm right mandibular positions without tooth contact while the examiner applied medially directed pressure against the patient’s chin for 20 seconds. Once all tests had been negative, the same examiner directly proceeded to bite registration procedures, beginning alternatively with BM or NM.

### Bimanual manipulation

Two layers of fused Beauty Pink X Hard wax (Miltex Inc., York, PA, USA) were trimmed to the buccal cusps and incisal edges of the maxillary dental arch to avoid interference with soft tissues. Another layer of fused wax covered the anterior third of the plate. Volunteers sat slightly reclined in a dental chair with their head comfortably supported by a headrest. A cotton roll, placed between the antagonist premolars, separated the dental arches for five minutes. Then the operator softened the wax, removed the cotton roll, and inserted the wax onto the air-syringe dried maxillary dental arch. As described by Dawson^3^, the operator placed 4 fingers to the posterior and inferior mandibular margin on each side and his thumbs lateral to the patient’s chin. The subject gently opened and closed the jaw without tooth contact 5 times. When the subject stopped, the operator continuously guided the open-close movements. Once no resistance was felt, he used firm upward pressure on the posterior mandibular edges while his thumbs pushed the chin downward. The wax recorded 0.5 to 1 mm shallow impressions of the teeth at approximately 2 mm vertical separation of the first molars. According to Dawson, the operator verified the BM position by instant repetition of the manipulation and checked if the second dental impressions fitted precisely into the first ones. All subjects showed clinically distinct repeatability.

### Neuromuscular bite registration

This technique recorded the point of unguided mandibular closure right before tooth contact. Same as for BM, individuals sat slightly reclined in a dental chair with a cotton roll between the premolars for 5 minutes. After removal of the cotton roll, subjects made a short maximum mouth opening, started closure but stopped just before antagonist tooth contact. Then they slowly opened and closed the mouth 10 times for 2–3 mm without tooth contact, made a 5-second break, and continued this mode for 2 minutes without any tooth contact at a speed of 20–30 strokes per minute. This unguided relaxed jaw movement produced the NM position, which was finally registered with polyvinyl siloxane (Blue Bite SC, Pluradent, Offenbach, Germany) from the left first molars to the right first molars at 2 mm vertical separation of the first molars. NM’s reproducibility was checked immediately by repeating the procedure. All participants regained the second NM position unimpededly. Using polyvinyl siloxane instead of wax helped avoid mix-up of bite registrations during the MRI examination.

### Magnetic resonance imaging

Right after the bite registrations each volunteer underwent MRI of both TMJs using a 3-Tesla scanner (Magnetom Skyra, Siemens, Erlangen, Germany). The head was positioned in a 16-channel head-neck coil. Restriction pads limited head movements during scanning. Subjects were instructed to avoid body movements. ICP was investigated without interocclusal bite registration. NM and BM condyle positions were scanned with intraorally repositioned polyvinyl siloxane and ice-water chilled wax bite registrations, respectively.

**Figure 3:**
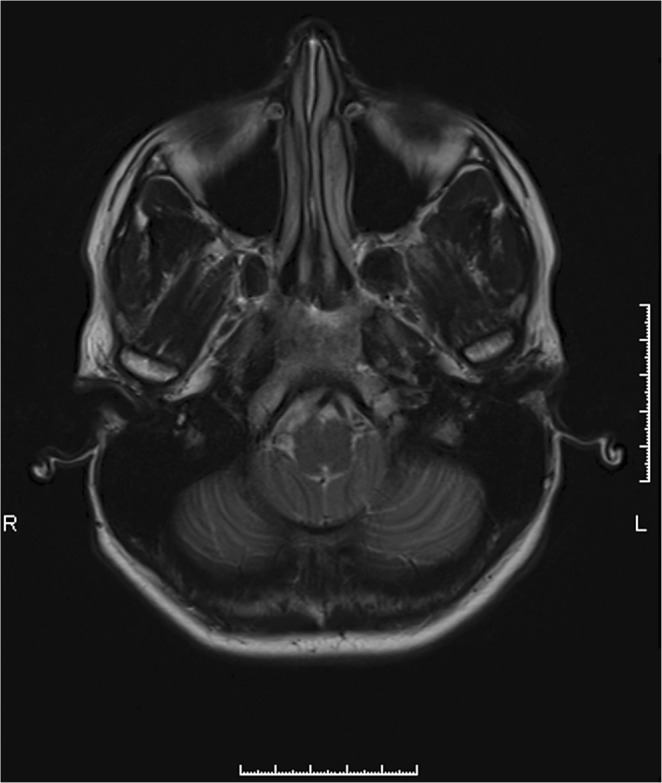
Axial view of the horizontal plane focusing optimum depiction of mandibular condyles. This MRI slice does not show the most superior condylar points used for measurements.

**Figure 4:**
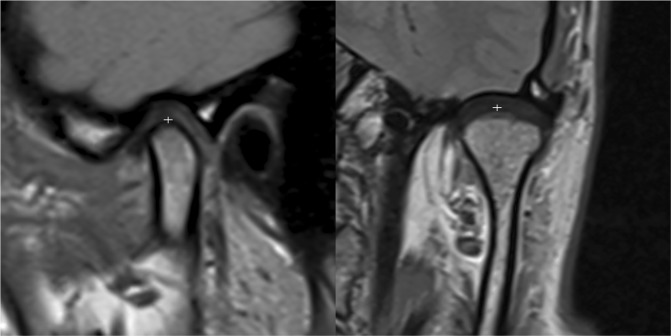
Proton-weighted MRIs showing most superior condylar points in sagittal (left) and coronal (right) planes.

Scans of 5.5 minutes each produced sagittal and coronal slices for a distinct jaw position. The sequences were proton weighted TSE images (2300 ms TR, 10 ms TE, flip angle 160°, distance factor 10%, averages 2, concatenation 1, band width 300 Hz/Px, matrix base resolution 320 Px, field of view 170 mm). Image resolution was 0.3 × 0.3 × 2 mm voxels. Dynamic imaging was not performed due to its reduced image resolution. Condyle position was detected using a transversal localizer scan (Fig. 3). Paracoronal slices (Fig. 4) ran along the condylar head axis. In anteroposterior direction, the sagittal slices were oriented perpendicular to the condylar head axis and parallel to the long axis of the ascending ramus.

Using Osirix medical imaging viewer (version MD; Pixmeo SARL, Geneva, Switzerland), we obtained XYZ-coordinates of measuring points at a magnification of 600%. Fifteen MRI slices of 2 mm slice increment depicted the position of each condyle. We selected the central parasagittal condylar slice for evaluation by bisection of the distance from the condyle’s medial pole to the lateral pole in half. We picked the slice nearest to the bisection. As shown in Fig. 4, we measured the most superior condylar points on central slices, beginning with ICP scans, then BM and NM on corresponding slices. All points were drawn three times by 2 operators in the frontal and parasagittal planes. Both operators simultaneously judged the measuring point site (4-eyes principle). We assessed the repeatability of landmark identification by measuring all ICP condyle points 3 and 6 days later and used Dahlberg’s formula to calculate errors. Inter- and intrarater reliabilities of ICP landmark identification were assessed using ICCs.

Anonymous XYZ data were transferred to an Excel file (Office 2016, Microsoft Corporation, Redmond, WA, USA) under surveillance of a second investigator. Subsequent comparisons of individual ICP, BM and NM positions originate from averaging 3 XYZ data sets of each position for each volunteer. Because of implicit inter-individual differences of setting the MRI coordinate system, we computed individual differences of the XYZ coordinates between ICP, BM, and NM positions for each subject and evaluated these differences for the total sample.

### Statistics

Descriptive and inferential statistics (α = 0.05), 95% predictive confidence ellipses of the data, and estimated 3D hulls for all positions were calculated using R software, version 3.5.2 (R Core Team 2018, Vienna, Austria). Confidence ellipses refer to Hotelling’s T^2^ for the data^31^. Superimposition of mirrored right-side data onto left side data yielded a point cloud, which represented a space of centric condyle positions. Hull volume calculations in mm^3^ refer to cloud shapes such as a minimal convex hull and a minimal ellipsoid hull. Confidence intervals for these volumes were estimated using bootstrap. Differences in projected planes as well as 3D Euclidean distances were modelled using generalized mixed models, including participant and side as random factors, type of measurement as independent factor, and Gaussian errors^32^. The model for Euclidean distances included a logarithmic link function to account for the right-skewedness of strictly positive distances. Results of all models are presented as estimated marginal means; tests and confidence intervals are based on the Kenward-Roger approximation^33^. We calculated ICCs and corresponding 95% CIs for presentation of intra- and interrater reliabilities of repeated measurements^34^.

### Ethical approval and informed consent

Approval: Authors confirm that all experimental protocols were approved by the Ethics Commission of the Medical University of Vienna (#ECS1438/2015).

Accordance: Authors confirm that methods were carried out according to the guidelines and regulations.

Informed consent was obtained from all participants.

## References

[1] Proffit, W. R., Fields, H. W. & Sarver, D. M. Contemporary orthodontics. 193 (Mosby 2007).

[2] Rinchuse DJ, Kandasamy S (2006). Centric relation: A historical and contemporary orthodontic perspective. J. Am. Dent. Assoc..

[3] Dawson PE (1973). Temporomandibular joint pain-dysfunction problems can be solved. J. Prosthet. Dent..

[4] Hobo S, Iwata T (1985). Reproducibility of mandibular centricity in three dimensions. J. Prosthet. Dent..

[5] McKee JR (1997). Comparing condylar position repeatability for standardized versus nonstandardized methods of achieving centric relation. J. Prosthet. Dent ..

[6] Brill N, Tryde G (1974). Physiology of mandibular positions. Front. Oral Physiol..

[7] Bumann, A. & Lotzmann, U. TMJ disorders and orofacial pain. 127 (Thieme 2002).

[8] Tripodakis AP, Smulow JB, Mehta NR, Clark RE (1995). Clinical study of location and reproducibility of three mandibular positions in relation to body posture and muscle function. J. Prosthet. Dent..

[9] Čelar A, Freudenthaler J, Crismani A, Graf A (2013). Guided and unguided mandibular reference positions in asymptomatic individuals. Orthod. Craniofac. Res..

[10] Alexander SR, Moore RN, DuBois LM (1993). Mandibular condyle position: Comparison of articulator mountings and magnetic resonance imaging. Am. J. Orthod. Dentofacial Orthop..

[11] Kandasamy S, Boeddinghaus R, Kruger E (2013). Condylar position assessed by magnetic resonance imaging after various bite position registrations. Am. J. Orthod. Dentofacial Orthop..

[12] Poveda Roda R, Bagan JV, Díaz Fernández JM, Hernández Bazán S, Jiménez Soriano Y (2007). Review of temporomandibular joint pathology. Part I: classification, epidemiology and risk factors. Med. Oral Patol. Cir. Bucal.

[13] Ogura I, Kaneda T, Mori S, Sakayanagi S, Kato M (2012). Magnetic resonance characteristics of temporomandibular joint disc displacement in elderly patients. Dentomaxillofac. Radiol..

[14] Yadav S, Yang Y, Dutra EH, Robinson JL, Wadhwa S (2018). Temporomandibular joint disorders in older adults. J. Am. Geriatr. Soc..

[15] Kanomi R, Hidaka O, Yamada C, Takada K (2004). Asymmetry in the condylar long axis and first molar rotation. J. Dent. Res..

[16] Toma AM (2012). The assessment of facial variation in 4747 British school children. Eur. J. Orthod..

[17] Ren YF, Isberg A, Westesson PL (1995). Condyle position in the temporomandibular joint. Comparison between asymptomatic volunteers with normal disk position and patients with disk displacement. Oral Surg. Oral Med. Oral Pathol. Oral Radiol. Endod..

[18] Shafagh I, Yoder JL, Thayer KE (1975). Diurnal variance of centric relation position. J. Prosthet. Dent..

[19] Linsen SS, Stark H, Samai A (2012). The influence of different registration techniques on condyle displacement and electromyographic activity in stomatognathically healthy subjects: a prospective study. J. Prosthet. Dent..

[20] Boos RH (1959). Vertical, centric and functional dimensions recorded by gnathodynamics. J. Am. Dent. Assoc..

[21] Chu SA, Suvinen TI, Clement JG, Reade PC (2001). The effect of interocclusal appliances on temporomandibular joints as assessed by 3D reconstruction of MRI scans. Aust. Dent. J..

[22] Lelis ER (2015). Cone-beam tomography assessment of the condylar position in asymptomatic and symptomatic young individuals. J. Prosthet. Dent..

[23] Kandasamy S, Greene CS, Obrez A (2018). An evidence-based evaluation of the concept of centric relation in the 21^st^ century. Quintessence Int..

[24] Graber, T. M. & Vanarsdall, R. L. Orthodontics: Current principles and techniques. 41 (Mosby 1994).

[25] Truitt J, Strauss RA, Best A (2009). Centric relation: a survey study to determine whether a consensus exists between oral and maxillofacial surgeons and orthodontists. J. Oral Maxillofac. Surg..

[26] Pullinger AG, Seligman DA, Gornbein JA (1993). A multiple logistic regression analysis of the risk and relative odds of temporomandibular disorders as a function of common occlusal features. J. Dent. Res..

[27] Raustia AM, Pirttiniemi PM, Pyhtinen J (1995). Correlation of occlusal factors and condyle position asymmetry with signs and symptoms of temporomandibular disorders in young adults. Cranio.

[28] Landi N, Manfredini D, Tognini F, Romagnoli F, Bosco M (2004). Quantification of the relative risk of multiple occlusal variables for muscle disorders of the stomatognathic system. J. Prosthet. Dent..

[29] Vieira-Queiroz I, Gomes Torres MG, de Oliviera-Santos C, Flores Campos PS, Crusoé-Rebello IM (2013). Biometric parameters of the temporomandibular joint and association with disc displacement and pain: a magnetic resonance imaging study. Int. J. Oral Maxillofac. Surg..

[30] Torres MGG, Crusoé-Rebello IM, Rosário M, Albuquerque MC, Flores Campos PS (2016). Morphometric features of the mandibular condyle and association with disk abnormalities. Oral Surg. Oral Med. Oral Pathol. Oral Radiol..

[31] Fox, J. An R and S-Plus companion to applied regression. (Sage 2002).

[32] Bates D, Mächler M, Bolker B, Walker S (2015). Fitting linear mixed-effects models using lme4. J. Stat. Softw..

[33] Halekoh U, Højsgaard S (2014). A Kenward-Roger approximation and parametric bootstrap methods for tests in linear mixed models – the R package pbkrtest. J. Stat. Softw..

[34] Rousson V, Gasser T, Seifert B (2002). Assessing intrarater, interrater and test-retest reliability of continuous measurements. Statist. Med..

